# Mitigating Oxidative Stress and Anti-Angiogenic State in an In Vitro Model of Preeclampsia by HY-12, an Organofluorine Hydrazone Antioxidant

**DOI:** 10.3390/cimb47090680

**Published:** 2025-08-24

**Authors:** Zsuzsanna K. Zsengellér, Maxim Mastyugin, Adrianna R. Fusco, Bernadett Vlocskó, Maximilian Costa, Coryn Ferguson, Diana Pintye, Réka Eszter Sziva, Saira Salahuddin, Brett C. Young, Marianna Török, Béla Török

**Affiliations:** 1 Department of Medicine, Division of Nephrology, Beth Israel Deaconess Medical Center, Boston, MA 02215, USA; maxim.mastyugin001@umb.edu (M.M.); adrianna.fusco001@umb.edu (A.R.F.); cfergus2@bidmc.harvard.edu (C.F.); dpintye@bidmc.harvard.edu (D.P.); 2 Department of Chemistry, University of Massachusetts Boston, Boston, MA 02125, USA; bernadett.vlocsko001@umb.edu (B.V.); max.costa001@umb.edu (M.C.); marianna.torok@umb.edu (M.T.); bela.torok@umb.edu (B.T.); 3 Department of OB/GYN, Semmelweis University, 1082 Budapest, Hungary; sziva.reka@semmelweis.hu; 4 Department of OB/GYN, Beth Israel Lahey Health, Boston, MA 02215, USA; ssalahud@bidmc.harvard.edu; 5 Department of OB/GYN, Mt Auburn Hospital, Boston, MA 02138, USA; brett.young@mah.harvard.edu

**Keywords:** preeclampsia, oxidative stress, organofluorine hydrazones, mitochondria, sFLT1, trophoblast cells, antioxidant therapy

## Abstract

Preeclampsia (PE) is a hypertensive disorder impacting 5–7% of pregnancies globally. With no causative treatment available, diagnosed patients have limited therapeutic options, putting them at risk for pregnancy complications. The induction of oxidative stress by ROS—one of the major contributors in PE pathogenesis—causes downstream signaling and production of anti-angiogenic factors, such as sFLT1 and sEng. The anti-angiogenic factors may cause endothelial and trophoblast dysfunction, contributing to the development of hypertension, proteinuria, and in severe cases, eclampsia. To target placental oxidative stress, we developed and evaluated an organofluorine hydrazone antioxidant, HY-12, in vitro. Human trophoblast (HTR8/SVneo) cells were incubated with hydrogen peroxide to induce oxidative stress and act as a model of PE. The goal of the study was to assess the efficacy of HY-12 and its ability to reduce cell injury, mitochondrial stress, and anti-angiogenic response. In our human trophoblast-based assays, pre-treatment with HY-12 reduced mitochondrial-derived ROS production in cells exposed to hydrogen peroxide, proving its ability to alleviate the oxidative stress associated with the pathogenesis of PE. HY-12 reduced HIF1A expression and sFLT1 protein expression in H_2_O_2_-exposed HTR8 cells. Furthermore, HY-12 improved the activity of the mitochondrial electron chain enzyme cytochrome C oxidase (COX) in the hydrogen-peroxide-treated HTR8/SVneo cells, which is a promising attribute of the compound. In reducing placental trophoblast oxidative stress, HY-12 shows promise as a potential treatment of preeclampsia. In vivo studies are warranted to further determine the efficacy of this compound.

## 1. Introduction

Preeclampsia (PE) is a serious complication characterized by hypertension and proteinuria occurring after 20 weeks of pregnancy [1,2]. It is associated with significant maternal, fetal and neonatal morbidity and mortality [3,4]. There is no cure available for PE. While etiology and pathogenesis are elusive, it is believed that placental ischemia, due to impaired spiral artery remodeling, is the primary cause [5]. Spiral artery remodeling occurs during normal implantation; extravillous trophoblasts invade the inner layer of the uterus and induce changes in the spiral arteries. These changes include obliteration of the tunica media that converts the arteries into low-pressure high-volume vessels, to sufficiently address the demands of the growing fetus. Improper remodeling, as seen in PE, leads to placental ischemia, reperfusion injury, and the release of reactive oxygen species (ROS) and reactive nitrogen species (RNS). The current hypothesis regarding this phenomenon is that the ischemic state contributes to oxidative stress, reducing endothelial and trophoblast function leading to PE [6,7,8,9,10,11,12,13]. Elevated levels of reactive oxygen species (ROS) and reactive nitrogen species (RNS) have also been observed in the placentas of patients with PE. Additionally, the activity of the mitochondrial electron transport chain enzyme cytochrome C oxidase (COX) declines in placental syncytiotrophoblast cells, depicting mitochondrial damage/dysfunction as a potential contributor to pathogenesis of PE [14,15,16,17,18]. Hypoxia-inducible factor 1 alpha (HIF1A) is a heteromeric protein complex that, under low oxygen tension, activates transcription of various anti-angiogenic factors such as soluble fms-like tyrosine kinase-1 (sFLT1) and soluble endoglin (sEng), and is stabilized by the presence of ROS and RNS [19,20,21,22]. sFLT1 and sEng bind to and decrease the levels of vascular endothelial growth factor (VEGF) and transforming growth factor beta, respectively. Therefore, when released in the bloodstream, these anti-angiogenic factors along with ROS/RNS lead to maternal endothelial dysfunction effecting multiple organs, culminating in hypertension, proteinuria, and other systemic manifestations of PE [23,24,25,26].

Since placental oxidative stress is hypothesized to be a major contributor to preeclampsia, it has been a target of drug development. Proposed therapeutics include antioxidants, such as Vitamin C and E. However, these studies have shown low efficacy in clinical trials [27,28,29,30,31] probably due to relatively low potency and poor mitochondrial uptake. Therefore, it is of interest to develop new antioxidants with improved pharmacodynamic and pharmacokinetic properties. Targeted antioxidants have been applied to models of ischemia–reperfusion injury [32,33,34] and proposed as an alternative treatment for the ischemia-induced oxidative stress found in preeclamptic tissue [15,17,35,36].

Although there are several natural small molecule antioxidants, such as polyphenols (e.g., resveratrol), available, their therapeutic success is usually limited due to delivery problems. Most of these antioxidants are overly polar to be able to pass through the cell membrane and be effective within the cell [37]. Since the discovery of the beneficial effects of fluorine incorporation into small molecule drugs on their biological properties [38], organofluorine drug development became a mainstream approach to enhance the membrane permeability of therapeutic candidates [39]. In fact, approximately 20% of all drugs on the market today contain at least one fluorine atom. Due to the high stability of the carbon trifluoride (CF_3_) group and aryl carbon–fluor (C-F) bond, such derivatives are particularly important substances in biomedical applications as they possess improved resistance to metabolic degradation. The increased lipophilicity of these compounds results in active agrochemical and pharmaceutical compounds with improved absorption, distribution, metabolism, and excretion (ADME) characteristics in vivo, enabling lower effective doses [40]. Organofluorine drugs are currently employed to treat a broad range of diseases, including roles as anticancer drugs, anesthetic agents, antidepressant drugs, and cardiovascular agents.

Learning from the early drug development of resveratrol-like compounds [41], we developed a novel series of diaryl hydrazones as multitarget agents for potential anti-Alzheimer’s therapeutic application [42,43]. In these works, the remarkable radical scavenging effect of these compounds had been established. Recently, we have extended the applications of diaryl hydrazones, primarily focusing on their antioxidant properties as potential mitochondrial antioxidant therapy agents to alleviate oxidative stress in preeclampsia and tested to determine whether these compounds reduce placental oxidative stress and lead to symptom mitigation in vitro [44]. The goal was to screen a broad variety of hydrazones and as a result, we have identified an organofluorine diaryl hydrazone (labeled as HY-12) as a lead compound.

In the current work, this HY-12 compound was selected and tested in a broad variety of cell biology assays using an in vitro preeclampsia model. We induced oxidative stress in human trophoblast (HTR8/SVneo; ATCC, Manassas, VA 20110, USA) cells with hydrogen peroxide (H_2_O_2_) and examined any changes in cell-redox function, cell injury, induction of the transcription factor HIF1A, and anti-angiogenic responses in cells treated with HY-12. These effects were compared to known antioxidants—Trolox, ascorbic acid, and MitoTEMPO. It was confirmed that HY-12 was superior to these reference compounds.

## 2. Materials and Methods

### 2.1. Radical Scavenging Assays

HY-12 and the reference antioxidants (ascorbic acid and Trolox) were dissolved in dimethyl sulfoxide (DMSO) to a concentration of 100 mM and a serial dilution was then performed to obtain compound stock solution concentrations at 50 mM, 25 mM, 6.25 mM, 3.23 mM, and 1.56 mM. The 2,2-diphenyl-1-picrylhydrazyl (DPPH) and 2,2’-azino-bis(3-ethylbenzo thiazoline-6-sulfonic acid (ABTS) assays were performed as described in our earlier article [45].

#### 2.1.1. DPPH Radical Scavenging Assay

The DPPH assay was conducted as described earlier [46]. For the DPPH assay, the compound stock solutions were diluted in 37 °C 50% ethanol by a factor of 50. DPPH (Sigma-Aldrich, St. Louis, MO, USA) was dissolved in 50 mL of 37 °C 50% ethanol to a concentration of 222 μM and stirred for 45 min in the dark to create the DPPH radical solution. On a clear flat bottom 96-well plate, 20 μL of each compound and 180 μL of DPPH were added in quadruplicate. Sets with 20 μL of 200 μM DMSO in 50% ethanol and 180 μL of 50% ethanol were used to determine the background absorbance. Sets with 20 μL of 200 μM DMSO in 50% ethanol and 180 μL of DPPH were used as the control group. The absorbance values were collected by a VersaMax UV-Vis plate reader that was set to 37 °C and 519 nm and processed with the SoftMax Pro 5 software (instrument and software are both from Molecular Devices LLC, San Jose, CA, USA). Readings were collected every 15 min for 60 min.

(1) $$Percent\;Radical\;Scavenging=\frac{\left({Abs}_{c}-{Abs}_{t}\right)}{{Abs}_{c}}\times 100$$

The 60 min values were standardized against the 60 min Trolox value to generate the Trolox equivalent values (Equation (2)).

(2) $$Trolox\;Equivalence=\frac{{Percent\;Radical\;Scavenging}_{Sample}\;}{{Percent\;Radical\;Scavenging}_{Trolox}}$$

#### 2.1.2. ABTS Radical Scavenging Assay

For the ABTS assay, 100 factor dilutions of the compound stock solutions were performed in ethanol. The ABTS radical solution was prepared 16 to 24 h before the assay was started by dissolving ABTS (Tokyo Chemical Industry, Tokyo, Japan) and K_2_S_2_O_8_ in 4 mL of deionized (DI) water to a concentration of 7 mM and 2.45 mM, respectively, and kept in the dark afterwards. The absorbance values were collected by a VersaMax UV-Vis plate reader that was set to 37 °C and 734 nm and processed with the SoftMax Pro 5 software (Molecular Devices). The ABTS radical solution was diluted with 37 °C 75 mM phosphate buffer with 75 mM NaCl at pH 7.4 until the absorbance was between 0.70 and 0.85 (the ratio was typically 5 μL of ABTS and 195 μL of phosphate buffer). On a clear flat-bottom 96-well plate, 4 μL of each compound and 196 μL of ABTS were added in triplicate. Sets with 4 μL of 500 μM DMSO in ethanol and 196 μL of phosphate buffer determined background absorbance. Sets with 4 μL of 500 μM DMSO in ethanol and 196 μL of ABTS were used as the control group. Absorbance readings were taken at 0, 6, and 12 min.

The data from both assays were processed using following equation (Equation (3)), where Abs_c_ is the absorbance of the control and Abs_t_ is the absorbance of the test sample.

(3) $$Percent\;Radical\;Scavenging=\frac{\left({Abs}_{c}-{Abs}_{t}\right)}{{Abs}_{c}}\times 100$$

The 12 min values were standardized against the 12 min Trolox value to generate the final Trolox equivalent values (Equation (2)).

#### 2.1.3. Statistical Analysis of Chemical Assays

Microsoft Excel (Redmond, WA, USA) was used to analyze the data. The percent radical scavenging values were calculated using the following equations (Equations (1)–(3)) to obtain the percent radical scavenging values for each compound at the final time point for each assay (12 min for DPPH, 60 min for ABTS) where Abs_c_ is the average absorbance of the control wells and Abs_t_ is the absorbance of the reference/experimental compound. The final time point percent radical scavenging values were standardized against the final time point percent radical scavenging of Trolox at the standard plate concentration (20 μM for DPPH, 10 μM for ABTS) using the following equation (Equation (2)) to find the Trolox equivalent values.

Data are presented as Mean ± Standard Deviation where the number of independent repeats is three (ABTS) or four (DPPH). The linear trendlines for the Trolox equivalent results of ascorbic acid, Trolox, and HY-12 for the DPPH and ABTS assays were plotted. The linear trendline equation along with the minimum and maximum activities were used to calculate the relative EC50 values. The minimum and maximum activities were at the lowest (3.13 μM for DPPH and 1.25 μM for ABTS) and highest (50 μM for DPPH and 20 μM for ABTS) plotted concentrations, respectively. The coefficient of determination of the linear trendlines were calculated using Microsoft Excel.

### 2.2. Biological Evaluations

#### 2.2.1. Cell Culture Studies

Human trophoblast HTR8/SVneo cell line was obtained from ATCC (American Type Culture Collection, Manassas, VA, USA). Cells were grown in RPMI supplemented with 5% fetal bovine serum and 1% penicillin-streptomycin in a humidified incubator containing 5% CO_2_ at 37 °C. Cells in the logarithmic growth phase were used in subsequent experimentation. Organofluorine antioxidant HY-12 was dissolved in DMSO and stored at −20 °C until usage. The final concentration of DMSO in the medium was kept at less than 0.1% to not influence cellular health. Cells that received hydrogen peroxide received a dosage of 100 μM. Cells in the HY-12 + H_2_O_2_-treated groups were pre-treated with 0.1–50 μM antioxidant for 30 min, respectively, and then they were treated with 100 μM H_2_O_2_ for 18 h in the media (containing hydrazones and hydrogen peroxide). At the end of the experiments, the culture supernatants were collected and stored at −20 °C until they were assayed.

#### 2.2.2. Cell Viability Assay

A CCK-8 test (Cell Counting Kit-8, Dojindo Molecular Technologies, Inc. Shanghai, China), was used to measure cell viability following hydrogen peroxide and antioxidant treatments. HTR8/SVneo cells were plated in 48-well culture plates for the 24 h prior to treatment. Following an 18 h treatment with an antioxidant and H_2_O_2_, a total of 10 μL CCK-8 reagent was added in and incubated for 1 h in a 5% CO_2_ incubator at 37 °C. After the incubation, optical density values were acquired at 450 nm using a microplate reader.

#### 2.2.3. Cell Biology Measurements

HTR8/SVneo cells were seeded in 48-well plates (Nalgene Nunc, Thermo Fisher Scientific, Waltham, MA, USA) and incubated at 37 °C in a 10% CO_2_ humidified incubator overnight. Next day, cells received H_2_O_2_ treatment for 18 h, along with various concentrations of HY-12 or reference antioxidant. After 18 h the cells were treated with MitoSOX™ Red (#M36008 Thermo Fisher Scientific, Waltham, MA, USA) fluorogenic dye at 25 nM and 5 nM final concentrations, respectively, for 15 min. Cells underwent three PBS washes prior to dye visualization. Fluorescence of the various dyes were visualized and photographed using an inverted EVOS^®^ FL Imaging System (Advanced Microscopy Group, Mill Creek, WA, USA).

#### 2.2.4. HIF1A Measurement

Immunofluorescence was used to determine the nuclear translocation of HIF1A. HTR-8/SVneo cells were treated as mentioned previously [47]. Following treatment, the cells underwent three PBS washes followed by 4% paraformaldehyde fixation. Samples were then permeabilized with 0.5% Triton X-100, blocked with 1% BSA and then incubated with anti-HIF1A antibody (Alexa Fluor^®^ 488 Anti-HIF1A antibody [EP1215Y] ABCAM#ab190197 1:200 dilution) overnight at 4 °C. DAPI was used to stain the nuclei. The coverslips were placed on glass slides and samples were viewed under an inverted EVOS^®^ FL Imaging System (Advanced Microscopy Group). Morphometric measurements of the trophoblasts were obtained through fluorescence microscopy images art 20× using MitoSOX™ Red and HIF1A and light microscopy images for trophoblasts COX EHC with an original magnification of 20×. Four images as replicates were used for analysis and measurements. Morphometric measurements were performed using ImageJ software version 1.47 (National Institute of Health [NIH], Bethesda, MD, USA; http://imagej.nih.gov/ij accessed on 1–30 June 2024). To determine staining intensity, the threshold was set to include the MitoSOX™ Red fluorescence product or 3,3’-diaminobenzidine (DAB) staining, and the mean intensity (optical density; OD) of reaction product was calculated per image. The mean intensity was divided by cell area volume to calculate positivity per area.

#### 2.2.5. sFLT-1 ELISA

Soluble FLT-1 (sFLT1) in culture medium was measured by ELISA using the human VEGF receptor 1 (VEGFR1) Quantikine kit (R&D Systems, Minneapolis, MN, USA) following the manufacturer’s instructions as previously described [15].

#### 2.2.6. COX In Situ Enzyme Chemistry

COX enzyme chemistry on HTR8/SVneo cells were performed as we published previously [44]. Representative digital images of cell preparations (Thermo Scientific™: Nunc™ Lab-Tek™ II Chamber Slide™ System) were obtained. Four images were acquired and quantified per sample as replicates. Morphometric measurements were performed as described previously in HIF1A measurements.

#### 2.2.7. Statistical Analysis of the Cell-Based Assays

GraphPad Prism 9.5 statistical software (San Diego, CA, USA) was used. After checking the normality tests (Kolmogorov–Smirnov, Saphiro–Wilk, D’Agostino & Pearson, and Anderson–Darling tests), in case of normal distribution, parametric unpaired T-test or analysis of variance (ANOVA) with Tukey’s post hoc test was used. Non-parametric Mann–Whitney U test or Kruskal–Wallis test with Dunn’s post hoc test was used when distribution was non-normal. Data are presented either in Mean ± Standard error of Mean (SEM) or in Median [Interquartile range/IQR]. Statistical significance was accepted when *p*-value was less than 0.05 (*p* < 0.05).

## 3. Results

### 3.1. Chemistry 

**Figure 1:**
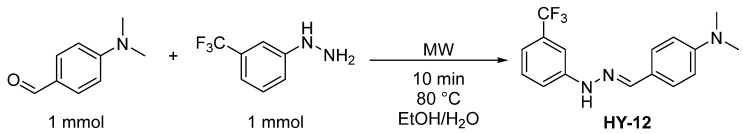
The schematic synthesis of HY-12.

We proposed that the unique combination of key structural features, such as the largely delocalized electron structure and the inclusion of fluorine in the structure, significantly contributed to the success of HY-12 as an effective antioxidant with excellent membrane permeability features, making it a viable option for PE drug development. In addition to the desirable characteristics of the compound, its synthesis is very simple and uses readily available commercial sources. The synthesis of HY-12 is outlined in Figure 1.

The compound was subjected to an in silico evaluation of its drug-like properties using SwissADME (http://www.swissadme.ch/ accessed on 15 March 2025) [48]. The calculations showed highly favorable characteristics for HY-12. The analysis revealed zero violation for the Lipinski- [49], Ghose- [50], Veber- [51], Egan- [52], and Muegge-selection criteria [53]. Other factors such as the PAINS screening are also advantageous for HY-12 as the compound generated zero PAINS alerts [54]. Further calculated pharmacokinetic factors, such as logP, also indicate a reasonable lipophilicity for HY-12. Depending on the method, its logP was found to be in the range of 2.78–5.18, and the consensus logP was determined to be 4.04. This is well within the range of compounds having practical druglike properties, such as balanced water solubility and membrane permeability. Also, its gastrointestinal absorption was characterized as high, which is promising for potential oral bioavailability. In addition, HY-12 has a low synthetic accessibility score of 2.65 (1 is easy, 10 is very difficult) indicating relatively simply achievable synthesis [45]. Considering that the cost of a drug is a major contributor to its commercial success, the easy synthesis is certainly a reasonable advantage. In addition, the compound is highly stable in a powder/crystalline form for an extended period (>1 year) [55].

### 3.2. Biochemistry

#### Radical Scavenging Activity of HY-12

We utilized the DPPH and ABTS assays to assess the antioxidant activity of HY-12 across a range of concentrations (3.13, 6.25, 12.5, 25, 50, and 200 μM for DPPH and 1.25, 2.5, 5, 10, and 20 μM for ABTS). In addition, reference antioxidants (ascorbic acid and Trolox) were also used to evaluate the comparative radical scavenging ability of HY-12. Ascorbic acid and Trolox, both broadly accepted as reference antioxidants, were chosen as controls due to their consistent results.

**Figure 2:**
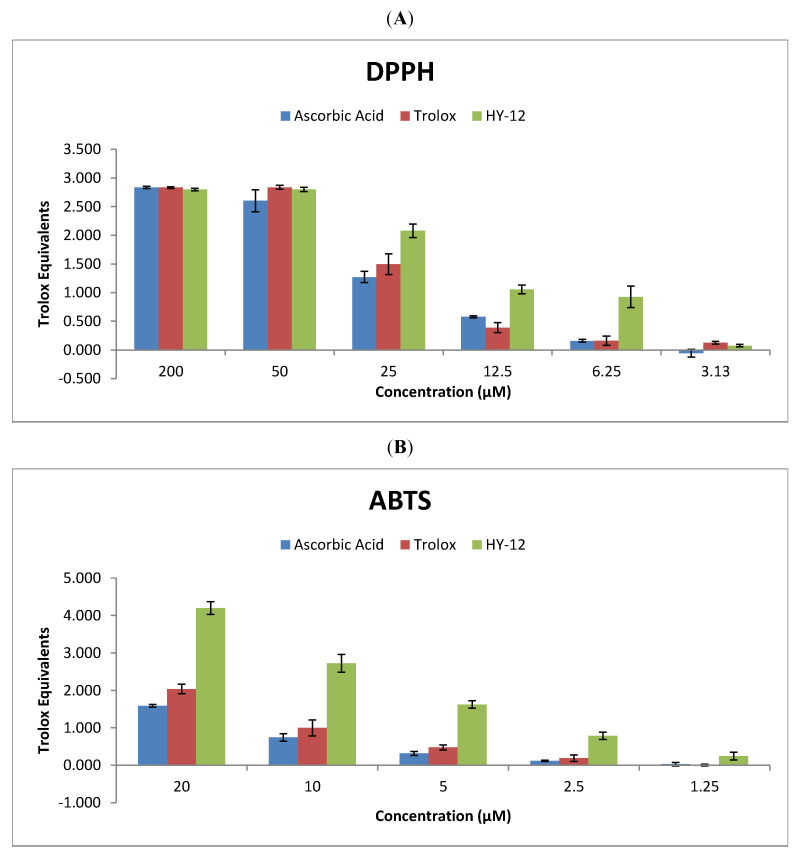
Radical scavenging potential of compound HY-12, used in this study, in comparison to Trolox and ascorbic acid. (**A**) Antioxidant activity measured in the DPPH (normalized to a 20 μM Trolox standard) and (**B**) ABTS assays (normalized to a 10 μM Trolox standard). The values are shown as mean of the Trolox equivalents ± standard deviation where the number of independent repeats is 3 (ABTS) or 4 (DPPH), respectively. The Trolox equivalent is calculated as the ratio of the percent radical scavenging activity of the test sample to the percent radical scavenging activity of the Trolox standard concentration listed at the individual assays above.

All percent radical scavenging values (calculated from absorbance intensities measured after 60 min for DPPH and 12 min for ABTS) were normalized to that of the standard plate concentration of Trolox for the respective assay (at the concentration of 20 μM Trolox for the DPPH and 10 μM Trolox for the ABTS assay). HY-12 was found to be a highly potent antioxidant as it had higher activity than the reference antioxidants in both assays, even showing activity at lower concentrations where the reference compounds were observed to be inactive (Figure 2).

The data clearly show that although at high concentrations the effect of HY-12 is nearly identical with those of ascorbic acid and Trolox, with decreasing antioxidant concentrations its superiority becomes unambiguous; at 6.25 μM concentration in the DPPH assay and 2.5 μM concentration in the ABTS assay, its activity is nearly 10 times that of the reference compounds, and at 1.25 μM in the ABTS assay it is still reasonably active while the reference compounds show no activity.

**Figure 3:**
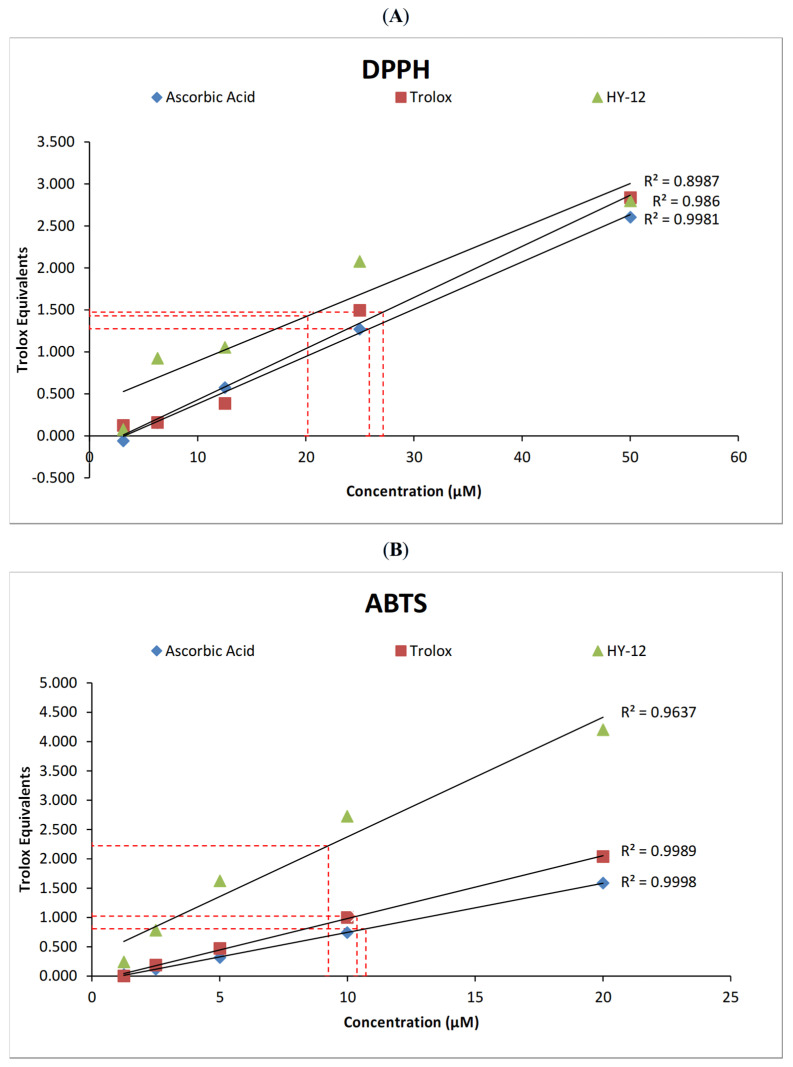
(**A**) Linear trendlines of the Trolox equivalents results of ascorbic acid, Trolox, and HY-12 in the DPPH and (**B**) in the ABTS assays. The relative EC_50_ (antioxidant concentration for 50% of the antioxidant’s maximum activity) of HY-12, as well as the reference compounds (ascorbic acid and Trolox) have also been determined to be 20.2 μM, 25.9 μM, and 27.1 μM, respectively, measured using the DPPH assay, and 9.25 μM, 10.7 μM, and 10.4 μM in the ABTS assay.

For more detailed characterization, the relative EC_50_ (antioxidant concentration required for reaching 50% of the antioxidant’s maximum activity) of HY-12, as well as those of the reference compounds, have been determined. The following values have been obtained: HY-12—20.2 μM, ascorbic acid—25.9 μM, and Trolox—27.1 μM in the DPPH assay (Figure 3A); and HY-12—9.25 μM, ascorbic acid—10.7 μM, and Trolox—10.4 μM in the ABTS assay (Figure 3B), with both sets being calculated as described in a previous paper [53]. In addition, the linear trendlines confirm the above observation. HY-12 shows better radical scavenging effect than the reference compounds at every concentration measured.

### 3.3. Cell Biology

#### 3.3.1. HY-12 Pre-Treatment Mitigated Mitochondrial-Derived Superoxide Production in H_2_O_2_-Exposed Trophoblast HTR8SVneo Cells

First, to achieve the optimized oxidative stress conditions, we examined HTR-8/SVneo cells treated with different concentrations of H_2_O_2_ (40, 60, 80, 100, and 120 μM) for 24 h, as described in a prior paper [47]. Cell viabilities were assessed by the CCK-8 assay. Cytotoxicity in HTR8/SVneo was dose-dependent, as higher cell deaths were associated with higher H_2_O_2_ concentrations. Our data suggests that the IC_50_ value of H_2_O_2_ concentration in this system was 100μM (50% killing of HTR-8/SVneo cells). This conclusion led to 100 μM H_2_O_2_ to be the baseline concentration for future experiments.

**Figure 4:**
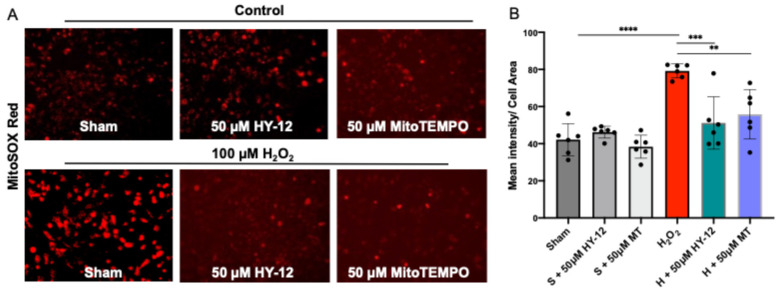
Compound HY-12 mitigated mitochondrial-derived superoxide release in H_2_O_2_-exposed trophoblasts cells (HTR8/SVneo). (**A**) Immunofluorescence analysis of Mito-SOX™ Red (superoxide production) in HTR8/SVneo cells treated with 50 μM HY-12 or 50 μM MitoTEMPO for 30 min, then exposed to 18 h H_2_O_2_. (Bars: 100 µm). (**B**) Quantitation of Mito-SOX™ Red immunofluorescence in trophoblasts: optical density per area (pixel2) of cell surface area was calculated in four high-power fields per sample (n = 5 per group). Mann–Whitney U test, Median [IQR]. ****: *p* < 0.0001, ***: *p* < 0.001, and **: *p* < 0.01.

In subsequent experiments, 100 μM H_2_O_2_ induced mitochondrial-derived superoxide production as measured by the MitoSox Red assay: the H_2_O_2_ group exhibited significantly higher intensity (*p* < 0.0001) than the control group. This was eliminated by pre-treatment with 50 µM HY-12 or MitoTEMPO, where there was significantly lower intensity compared to H_2_O_2_-treated group. HY-12 caused decreases similarly to MitoTEMPO (Figure 4).

Our data suggests that mitochondrial oxidative stress (superoxide—O_2_^·-^ measured by MitoSOX Red) is observed in our H_2_O_2_-induced cell culture model and it can be reduced by pre-treatment with HY-12. This is significant since oxidative stress is a key component in the etiology of preeclampsia.

#### 3.3.2. HY-12 Pre-Treatment Normalized HIF1A Expression in H_2_O_2_-Exposed Trophoblast HTR8/SVneo Cells

**Figure 5:**
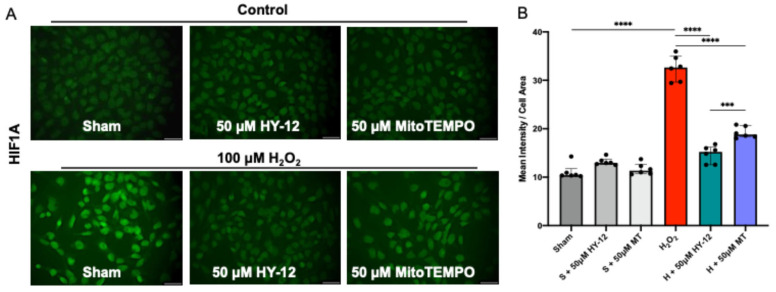
Compound HY-12 normalized expression of HF1A in H_2_O_2_-exposed trophoblast cells (HTR8/SVneo): (**A**) Immunofluorescence analysis of HIF1A expression in HTR8/SVneo cells treated with 50 μM HY-12 or 50 μM MitoTEMPO for 30 min, then exposed to 18 h H_2_O_2_. (Bars: 100 µm). (**B**) Quantitation of HIF1A immunofluorescence in trophoblast cells: optical density per area (pixel^2^) of cell surface area was calculated in four high-power fields per sample (n = 5 per group). Mann–Whitney U test, Median [IQR]. ****: *p* < 0.0001, and ***: *p* < 0.001.

In the next step, we assessed whether the H_2_O_2_-induced oxidative stress upregulated the transcription factor HIF1A in these cells. The results are shown in Figure 5.

In H_2_O_2_-induced cells, significantly increased green fluorescence intensity (HIF1A) can be seen compared to control (*p* < 0.0001). Pre-treatment of the cells with antioxidant HY-12 significantly reduced HIF1A expression (*p* < 0.0001), and achieved it more efficiently than MitoTEMPO, the reference antioxidant (*p* < 0.001) (Figure 4).

#### 3.3.3. HY-12 Pre-Treatment Diminished sFLT1 Protein in H_2_O_2_-Exposed Trophoblast HTR8/SVneo Cells

**Figure 6:**
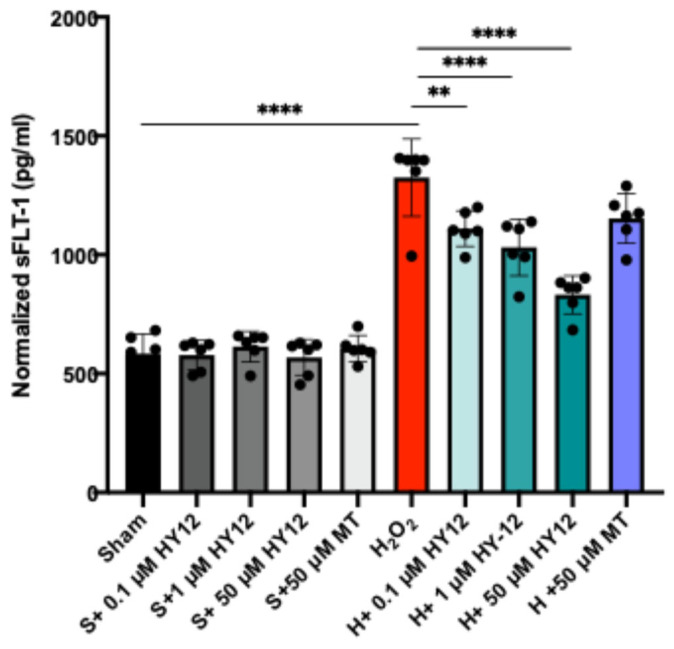
Compound HY-12 diminished sFLT1 protein expression in H_2_O_2_-exposed trophoblast cells (HTR8/SVneo). Normalized sFLT1 data according to different treatment groups (n = 4 per group). Mann–Whitney U test. Median [IQR]. ****: *p* < 0.0001, and **: *p* < 0.01.

The expression of the anti-angiogenic factor sFLT1 was also assessed in the H_2_O_2_ stressed HTR8/SVneo cells. Increased serum sFLT-1 concentration is related to the development of PE; indeed, the detection of significantly higher than normal sFLT-1 levels can forecast the diagnosis of PE 5 weeks prior to the clinical symptoms. In fact, the US FDA recently approved a blood test that identifies at-risk pregnant women, and thus preventative measures could be applied against PE [56,57]. The data are shown in Figure 6.

As expected, the stressed cells exhibited elevated sFLT1 production as assessed by ELISA, and HY-12 pre-treatment dose-dependently reduced the production of sFLT1 (Figure 6). This is a significant result since sFLT1 is known to induce the cardinal features of preeclampsia in vivo. To substantiate our findings, we isolated human villous explants and exposed them to hypoxia which resulted in increased sFLT1 expression in the culture supernatant. HY-12 pre-treatment dose-dependently reduced sFLT1 expression in the hypoxia-exposed explants (Supplementary Figure S6).

#### 3.3.4. HY-12 Pre-Treatment Improved Mitochondrial COX Activity in H_2_O_2_-Exposed Trophoblast HTR8/SVneo Cells

**Figure 7:**
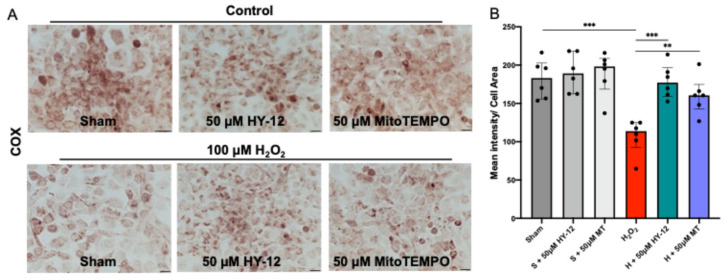
Compound HY-12 enhanced mitochondrial cytochrome C activity in H_2_O_2_-exposed trophoblast cells (HTR8/SVneo): (**A**) COX (cytochrome C oxidase) enzyme histochemistry in HTR8/SVneo cells treated with 50 μM HY-12 or 50 μM MitoTEMPO (MT) for 30 min, then exposed to 18 h H_2_O_2_. (Bars: 100 µm). Brown color indicates the COX enzyme activity. (**B**) Quantitation of COX enzyme activity in trophoblast cells: optical density per area (pixel^2^) of cell surface area was calculated in four high-power fields per sample (n = 5 per group). Mann–Whitney U test, Median [IQR]. ***: *p* < 0.001, **: *p* < 0.01.

As the production of ROS, i.e., the generation of oxidative stress, mainly occurring in the mitochondria, the electron transport chain activity was evaluated by mitochondrial cytochrome coxidase (COX) enzyme chemistry as shown in Figure 7.

It was observed that the HY-12 addition significantly decreased the enzyme activity in response to H_2_O_2_-treatment (*p* < 0.01) (Figure 7B). COX activity was also restored by MitoTEMPO pre-treatment, with similar efficiency to that of HY-12 (Figure 7A,B).

## 4. Discussion

Preeclampsia (PE) is a multi-system disorder with oxidative stress being established as a key initiating factor by us and others [6,7,9,11,15,17,35,57,58,59,60]. This study aimed to test whether a newly developed organofluorine hydrazone HY-12 can alleviate PE symptoms in an in vitro model. We found that HY-12 could lower oxidative stress and the development of downstream pathways, making it an exciting new treatment paradigm for this complex disease.

Varying enzyme systems exist to control the redox status in cells and tissues, including superoxide dismutase, glutathione reductase, and catalase in addition to endogenous (e.g., urea, melatonin) or exogenous/dietary small molecule free-radical scavengers, (e.g., resveratrol) [29,56,57], but these can be overwhelmed or depleted in cases of severe oxidative stress. In such cases, additional supplemental antioxidants can help restore redox homeostasis, prevent peroxidation of proteins and other cellular components, thereby maintaining cellular integrity and health. Unfortunately, Vitamin C and E supplementation during preeclamptic pregnancies has not been beneficial [29,31]. We therefore sought to employ other classes of antioxidants such as the organofluorine hydrazones. In the present study, HY-12 organofluorine hydrazone reduced oxidative stress with similar or better efficiency than mitochondrial-targeted antioxidant MitoTEMPO.

We also found that HIF1A, a transcription factor for anti-angiogenic factors, has been reduced by HY-12 treatment in trophoblast cells subjected to H_2_O_2_. Furthermore, sFLT1 expression has been reduced in this experimental setting which also contributes to the fact that HIF1A can upregulate this anti-angiogenic factor in human preeclampsia as well. Vascular endothelial growth factor (VEGF) and placental growth factor (PlGF) antagonize the antiangiogenic effects of sFLT1. The main effects of sFLT1 are vasoconstriction and endothelial damage, contributing to preeclampsia and a reduction in fetal development. The sFLT1/PlGF ratio is now a predictive and actionable biomarker for preeclampsia [61,62]. This ratio is based on the levels of sFLT1 and PlGF in maternal circulation. A high sFlt-1/PlGF ratio correlates to a higher risk of developing preeclampsia. Therefore, the use of HY-12 could be very useful in preventing anti-angiogenic response which needs further testing in in vivo models of PE.

Another positive outcome of HY-12 administration was the improved mitochondrial function which is reduced in PE as shown by us and others [14,15]. This phenomenon is commonly tested by measuring cytochrome C oxidase activity using enzyme histochemistry of the trophoblast cells, a functional assay. We have used this measurement in the past and also in the tissue section from human placentas which has shown low COX enzyme activity. Therefore, HY-12 improving COX activity is a major finding and shows an added benefit of the use of this drug candidate to alleviate the symptoms of PE.

Based on the above data, it is unambiguous that compound HY-12 showed significant promise in alleviating oxidative stress in in vitro models, such as radical scavenging of two organic radicals and in the human trophoblast cell-based vitro model of PE. The further development of the project includes the structure–activity relationship of HY-12 derivatives, pharmacokinetic studies on the in vivo behavior of HY-12 as well as the in vivo validation of the PE alleviating effect of HY-12 in the murine model of preeclampsia.

## 5. Conclusions

In conclusion, the organofluorine hydrazone HY-12 shows therapeutic promise through its ability to reduce trophoblast oxidative stress and enhance mitochondrial function in vitro. These characteristics make it of interest for the treatment of preeclampsia in which oxidative stress drives pathogenesis. Thus, this data supports the testing of HY-12 in a relevant in vivo model of preeclampsia.

## Data Availability

The original contributions/data presented in this study are included in the article and the supplementary material. Further inquiries can be directed to the corresponding author. Raw data from the assays have been deposited in the Open Science Framework (Link to repository data: https://osf.io/t6uje/files/osfstorage, accessed on 12 August 2025.

## Supplementary Materials

The following supporting information can be downloaded at: https://www.mdpi.com/article/10.3390/cimb47090680/s1.
