# Correction: Zsengellér et al. Mitigating Oxidative Stress and Anti-Angiogenic State in an In Vitro Model of Preeclampsia by HY-12, an Organofluorine Hydrazone Antioxidant. *Curr. Issues Mol. Biol.* 2025, *47*, 680

**DOI:** 10.3390/cimb48030301

**Published:** 2026-03-11

**Authors:** Zsuzsanna K. Zsengellér, Maxim Mastyugin, Adrianna R. Fusco, Bernadett Vlocskó, Maximilian Costa, Coryn Ferguson, Diana Pintye, Réka Eszter Sziva, Saira Salahuddin, Brett C. Young, Marianna Török, Béla Török

**Affiliations:** 1 Department of Medicine, Division of Nephrology, Beth Israel Deaconess Medical Center, Boston, MA 02215, USA; maxim.mastyugin001@umb.edu (M.M.); adrianna.fusco001@umb.edu (A.R.F.); cfergus2@bidmc.harvard.edu (C.F.); dpintye@bidmc.harvard.edu (D.P.); 2 Department of Chemistry, University of Massachusetts Boston, Boston, MA 02125, USA; bernadett.vlocsko001@umb.edu (B.V.); max.costa001@umb.edu (M.C.); marianna.torok@umb.edu (M.T.); bela.torok@umb.edu (B.T.); 3 Department of OB/GYN, Semmelweis University, 1082 Budapest, Hungary; sziva.reka@semmelweis.hu; 4 Department of OB/GYN, Beth Israel Lahey Health, Boston, MA 02215, USA; ssalahud@bidmc.harvard.edu; 5 Department of OB/GYN, Mt Auburn Hospital, Boston, MA 02138, USA; brett.young@mah.harvard.edu

In the original publication [1], there were mistakes in “2.1.3. Statistical Analysis of Chemical Assays”, “3.2 Biochemistry”, and Figures 2 and 3 as published. These mistakes were made because the authors did not consider the final dilution of the stock solution in the plate (10-fold dilution for the DPPH assay and 50-fold dilution for the ABTS assay) when calculating and plotting the concentration values. The corrections to the text in Figure 2 and Figure 3 appear below.

**Text Correction**: There were the following errors in the original publication. The sentence “The final time point percent radical scavenging values were standardized against the final time point percent radical scavenging of Trolox at the standard plate concentration (200 μM for DPPH, 500 μM for ABTS) using the following equation (Equation (2)) to find the Trolox equivalent values.” in Section 2.1.3, in the first paragraph, refers to two incorrect numbers: (200) μM for DPPH and (500) μM for ABTS. A correction has been made to this sentence so that the updated sentence now reads as “The final time point percent radical scavenging values were standardized against the final time point percent radical scavenging of Trolox at the standard plate concentration (20 μM for DPPH, 10 μM for ABTS) using the following equation (Equation (2)) to find the Trolox equivalent values.”

The sentence “The minimum and maximum activities were at the lowest (31.3 μM for DPPH and 62.5 μM for ABTS) and highest (500 μM for DPPH and 1000 μM for ABTS) plotted concentrations, respectively.” in Section 2.1.3, in the last paragraph, refers to four incorrect numbers: (31.3) μM for DPPH, (62.5) μM for ABTS, (500) μM for DPPH, and (1000) μM for ABTS. A correction has been made to this sentence so that the updated sentence now reads as “The minimum and maximum activities were at the lowest (3.13 μM for DPPH and 1.25 μM for ABTS) and highest (50 μM for DPPH and 20 μM for ABTS) plotted concentrations, respectively.”

The sentence “We utilized the DPPH and ABTS assays to assess the antioxidant activity of HY-12 across a range of concentrations (31.3, 62.5, 125, 250, 500, and 2000 μM for DPPH and 62.5, 125, 250, 500, and 1000 μM for ABTS).” in Section 3.2, in the first paragraph, refers to 11 incorrect numbers: 31.3, 62.5, 125, 250, 500, and 2000 μM for DPPH and 62.5, 125, 250, 500, and 1000 μM for ABTS. A correction has been made to this sentence so that the updated sentence now reads as “We utilized the DPPH and ABTS assays to assess the antioxidant activity of HY-12 across a range of concentrations (3.13, 6.25, 12.5, 25, 50, and 200 μM for DPPH and 1.25, 2.5, 5, 10, and 20 μM for ABTS).”

The sentence “All percent radical scavenging values (calculated from absorbance intensities measured after 60 min for DPPH and 12 min for ABTS) were normalized to that of the standard working solution of Trolox for the respective assay (at the concentration of 200 μM Trolox for the DPPH and 500 μM Trolox for the ABTS assay).” in Section 3.2, in the second paragraph, refers to two incorrect numbers and an incorrect word: (200) μM Trolox, (500) μM, and “working”. This sentence has been updated to “All percent radical scavenging values (calculated from absorbance intensities measured after 60 min for DPPH and 12 min for ABTS) were normalized to that of the standard plate concentration of Trolox for the respective assay (at the concentration of 20 μM Trolox for the DPPH and 10 μM Trolox for the ABTS assay).”

The sentence “The data clearly show that although at high concentrations the effect of HY-12 is nearly identical with those of ascorbic acid and Trolox, with decreasing antioxidant concentrations its superiority becomes unambiguous; at 125 μM concentration its activity is nearly 10 times that of the reference compounds, and at 62.5 μM it is still reasonably active while the reference compounds show no activity.” in Section 3.2, refers to two incorrect numbers: (125) μM and (62.5) μM. This sentence has been updated to “The data clearly show that although at high concentrations the effect of HY-12 is nearly identical with those of ascorbic acid and Trolox, with decreasing antioxidant concentrations its superiority becomes unambiguous; at 6.25 μM concentration in the DPPH assay and 2.5 μM concentration in the ABTS assay, its activity is nearly 10 times that of the reference compounds, and at 1.25 μM in the ABTS assay it is still reasonably active while the reference compounds show no activity.”

The sentence “The following values have been obtained: HY-12—202 μM, ascorbic acid—259 μM, and Trolox—271 μM in the DPPH assay (Figure 3A); and HY-12—463 μM, ascorbic acid—537 μM, and Trolox—519 μM in the ABTS assay (Figure 3B), with both sets being calculated as described in a previous paper [53].” in Section 3.2, refers to six incorrect numbers: (202), (259) μM, (271) μM, (463) μM, (537) μM, and (519) μM. This sentence has been updated to “The following values have been obtained: HY-12—20.2 μM, ascorbic acid—25.9 μM, and Trolox—27.1 μM in the DPPH assay (Figure 3A); and HY-12—9.25 μM, ascorbic acid—10.7 μM, and Trolox—10.4 μM in the ABTS assay (Figure 3B), with both sets being calculated as described in a previous paper [53].”

**Figure corrections:**

**Figure 2:**
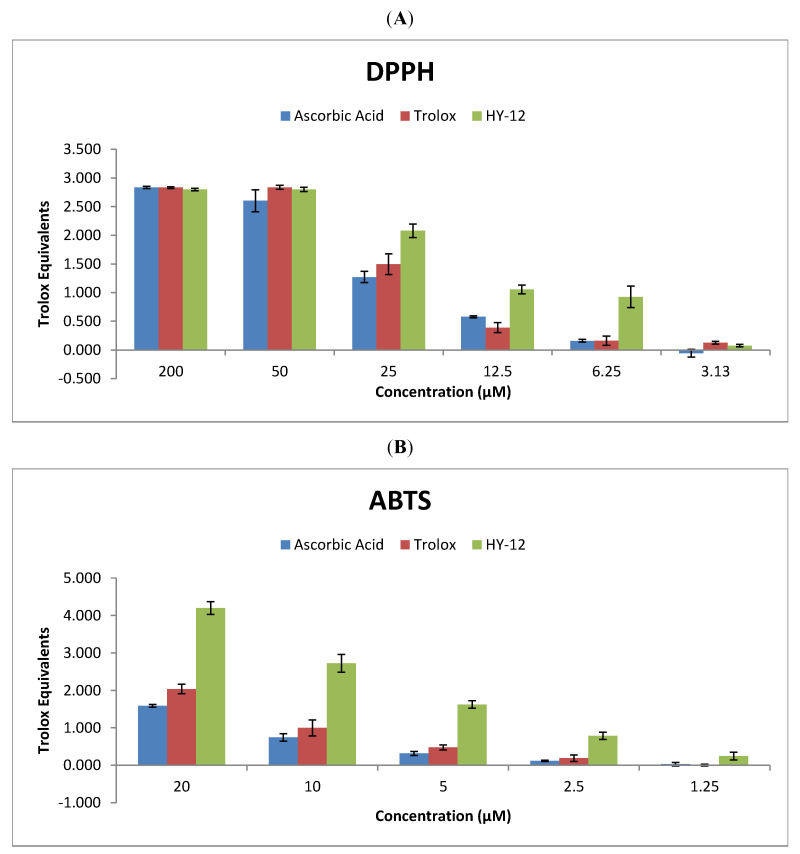
Radical scavenging potential of compound HY-12, used in this study, in comparison to Trolox and ascorbic acid. (**A**) Antioxidant activity measured in the DPPH (normalized to a 20 μM Trolox standard) and (**B**) ABTS assays (normalized to a 10 μM Trolox standard). The values are shown as mean of the Trolox equivalents ± standard deviation where the number of independent repeats is 3 (ABTS) or 4 (DPPH), respectively. The Trolox equivalent is calculated as the ratio of the percent radical scavenging activity of the test sample to the percent radical scavenging activity of the Trolox standard concentration listed at the individual assays above.

**Figure 3:**
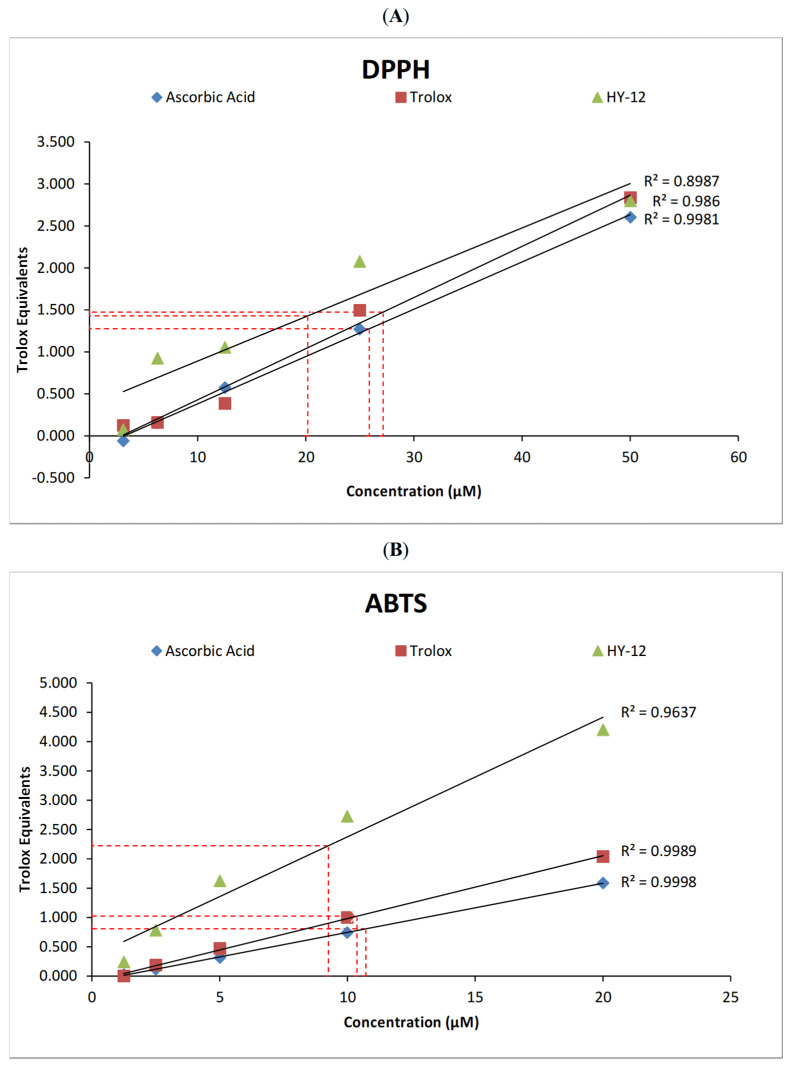
(**A**) Linear trendlines of the Trolox equivalents results of ascorbic acid, Trolox, and HY-12 in the DPPH and (**B**) in the ABTS assays. The relative EC_50_ (antioxidant concentration for 50% of the antioxidant’s maximum activity) of HY-12, as well as the reference compounds (ascorbic acid and Trolox) have also been determined to be 20.2 μM, 25.9 μM, and 27.1 μM, respectively, measured using the DPPH assay, and 9.25 μM, 10.7 μM, and 10.4 μM in the ABTS assay.

The authors state that the scientific conclusions are unaffected. This correction was approved by the Academic Editor. The original publication has also been updated.
