# Supplementary material for: Mitigating Oxidative Stress and Anti-Angiogenic State in an In Vitro Model of Preeclampsia by HY-12, an Organofluorine Hydrazone Antioxidant
Source: Curr Issues Mol Biol. 2025 Aug 24;47(9):680. doi: 10.3390/cimb47090680 (PMC12468959; doi:10.3390/cimb47090680)
Supplement: Supplementary file 1 [file cimb-47-00680-s001.zip › cimb-3790366-supplementary.pdf]

SUPPORTING INFORMATION

to

Mitigating oxidative stress and anti-angiogenic state in an in vitro model of preeclampsia by HY-12 an organofluorine hydrazone antioxidant

by Z. K. Zsengellér [1]*, M. Mastyugin[1,2], A. R. Fusco[1,2], R. B. Vlocsko[2], M. Costa[2], C. Ferguson[1], D. Pintye[1], R. E. Sziva[3], S. Salahuddin[4], B. C. Young[5], M. Török[2], and B. Török [2]

[1] *Department of Medicine, Beth Israel Lahey Health, Boston MA, USA*
[2] *Department of Chemistry, University of Massachusetts Boston, Boston MA, USA*
[3] *Department of OB/GYN, Semmelweis University, Budapest, Hungary*
[4] *Department of OB/GYN, Beth Israel Lahey Health, Boston MA, USA*
[5] *Department of OB/GYN, Mt Auburn Hospital, Boston MA, USA*
**Correspondence:* zzsengel@bidmc.harvard.edu

Table of Contents

1. Chemistry …… S2
1.1. General Information …… S2
1.2. Synthesis of HY-12 …… S2
1.3. Spectral data of HY-12 …… S2
1.4. HPLC-MS trace of HY-12 …… S6
1.5. Human Placental Villous Explant Cultures and Sample Collection …… S6
2. Biochemistry …… S7
2.1. Radical scavenging assays …… S7
2.1.1. DPPH radical scavenging assay …… S7
2.1.2. ABTS radical scavenging assay …… S8
3. Biological Evaluation …… S8
3.1. Cell Culture Studies …… S8
3.2. Cell Viability Assay …… S9
3.3. Cell Biology Measurements …… S9
3.4. HIF1A Measurements …… S10
3.5. Human Placental Villous Explant Cultures and Sample Collection …… S10
3.6. sFLT1-1 ELISA …… S10
3.7. COX in situ Enzyme Chemistry …… S10
3.8. Statistical Analysis …… S11
4. References …… S11

1. Chemistry:

1.1. General Information The starting materials for the synthesis of **HY-12** (4-N,N-dimethylbenzaldehyde and 3-trifluoromethyl-phenylhydrazone), the NMR reference compounds were all acquired from Aldrich. The NMR solvents, DMSO-d6 and $CDCl_3$ (99.8%), were obtained from Sigma-Aldrich and Cambridge Isotope Laboratories. Additional materials and supplies were acquired from Fisher Scientific.

The identification and purity of the product **HY-12** was determined by various mass spectrometric methods (GC-HRMS, HPLC-MS) and ^{1}H, ^{13}C and ^{19}F NMR spectroscopy. High-resolution mass spectrometry (HRMS) measurements were conducted using an Agilent 7250 GC-QTOF mass spectrometer, also in electron impact ionization (EI, 70 eV) mode. The HPLC-MS analysis was carried out by an Agilent 1260 Infinity II Ultivo LC/QQQ system. The ^{1}H, ^{13}C, and ^{19}F NMR spectra were recorded on a 400 MHz Agilent MM2 NMR spectrometer using DMSO-d_6 as a solvent. Tetramethylsilane (TMS) was used as internal standards, or the residual solvent signal served as a reference. Chemical shifts (δ) are reported in ppm. The following abbreviations are used to denote signal multiplicity in ^{1}H NMR spectra: s (singlet), d (doublet), t (triplet), q (quartet), and m (multiplet). All measurements were conducted at 25 °C with a temperature accuracy of ±1 °C.

1.2. Synthesis of the HY-12

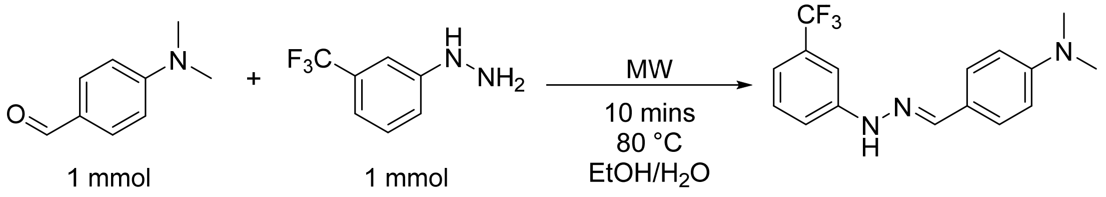

Scheme S1. Synthesis of the diaryl hydrazone, **HY-12**.

4-(Dimethylamino)-benzaldehyde (1.0 mmol) and 3-trifluoromethyl-phenylhydrazine (1.0 mmol) (or its commercially available HCl salt) were combined in 2 mL of ethanol, followed by the addition of 1 mL of water. The mixture was stirred under microwave (MW) irradiation at 80 °C for 10 min at standard energy input of 250 W. Upon completion, the reaction mixture was cooled in the freezer for 15 mins. The obtained precipitate was collected by vacuum filtration and let to

air dry. No further purification was needed. The identification was based on high resolution mass spectrometry (HRMS), ^{1}H, ^{13}C and ^{19}F NMR and the purity was verified by using GC−MS, and HPLC-MS.

1.3. Spectral data of HY-12:

(E)-N,N-dimethyl-4-((2-(3-(trifluoromethyl)phenyl)hydrazinylidene)methyl)aniline (**HY-12**)

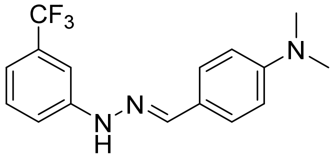

^{1}H NMR (399.822 MHz, DMSO-d_6) δ (ppm): 10.42 (s, 1H), 7.80 (s, 1H), 7.48-7.45 (m, 4H), 7.09-7.07 (d, 2H), 6.71-6.69 (d, 2H), 2.91 (s, 6H); **^{13}C NMR** (DMSO-d_6, 100 MHz) δ (ppm): 150.66, 149.46, 139.68, 130.54-129.61 (q, J=32 Hz), 130.15, 128.65-120.52 (q, J=271 Hz), 127.28, 123.10, 115.30, 113.86-113.82 (q, J=4 Hz), 112.10, 107.41-107.37 (q, J=4 Hz), 39.93; **^{19}F NMR** (376 MHz, DMSO-d_6) δ (ppm): -61.43; **HRMS** (EI): m/z: [M+] calcd. for $C_{16}H_{16}F_3N_3$ 307.1296; found: 307.1323.

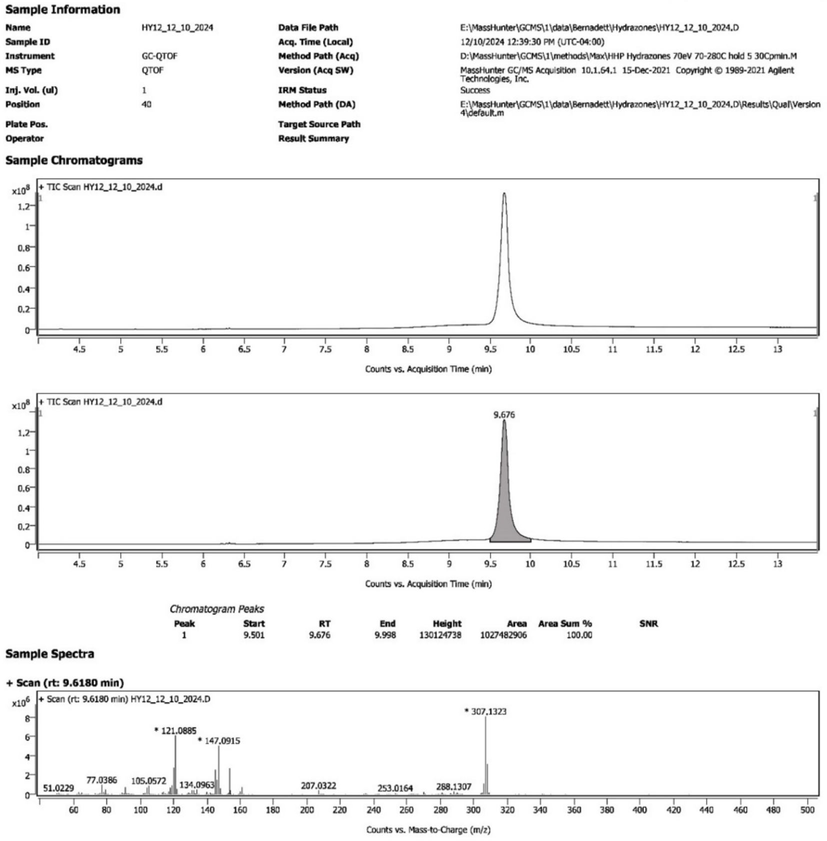

Figure S1 HRMS spectrum of (*E*)-*N,N*-dimethyl-4-((2-(3-(trifluoromethyl)phenyl)-hydrazinylidene)-methyl) aniline (**HY-12**).

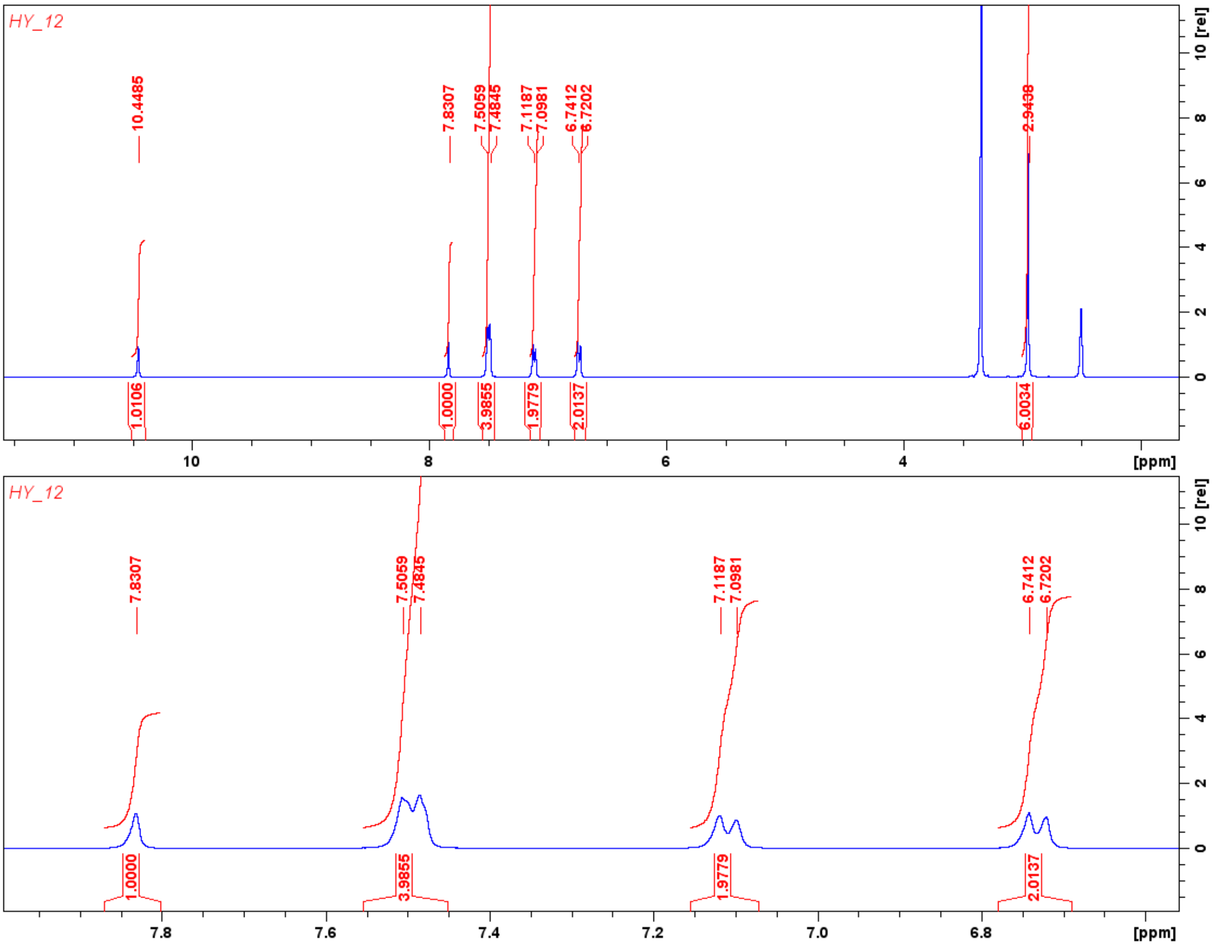

Figure S2 ^{1}H NMR of (*E*)-*N,N*-dimethyl-4-((2-(3-(trifluoromethyl)phenyl)hydrazinylidene)-methyl) aniline (**HY-12**).

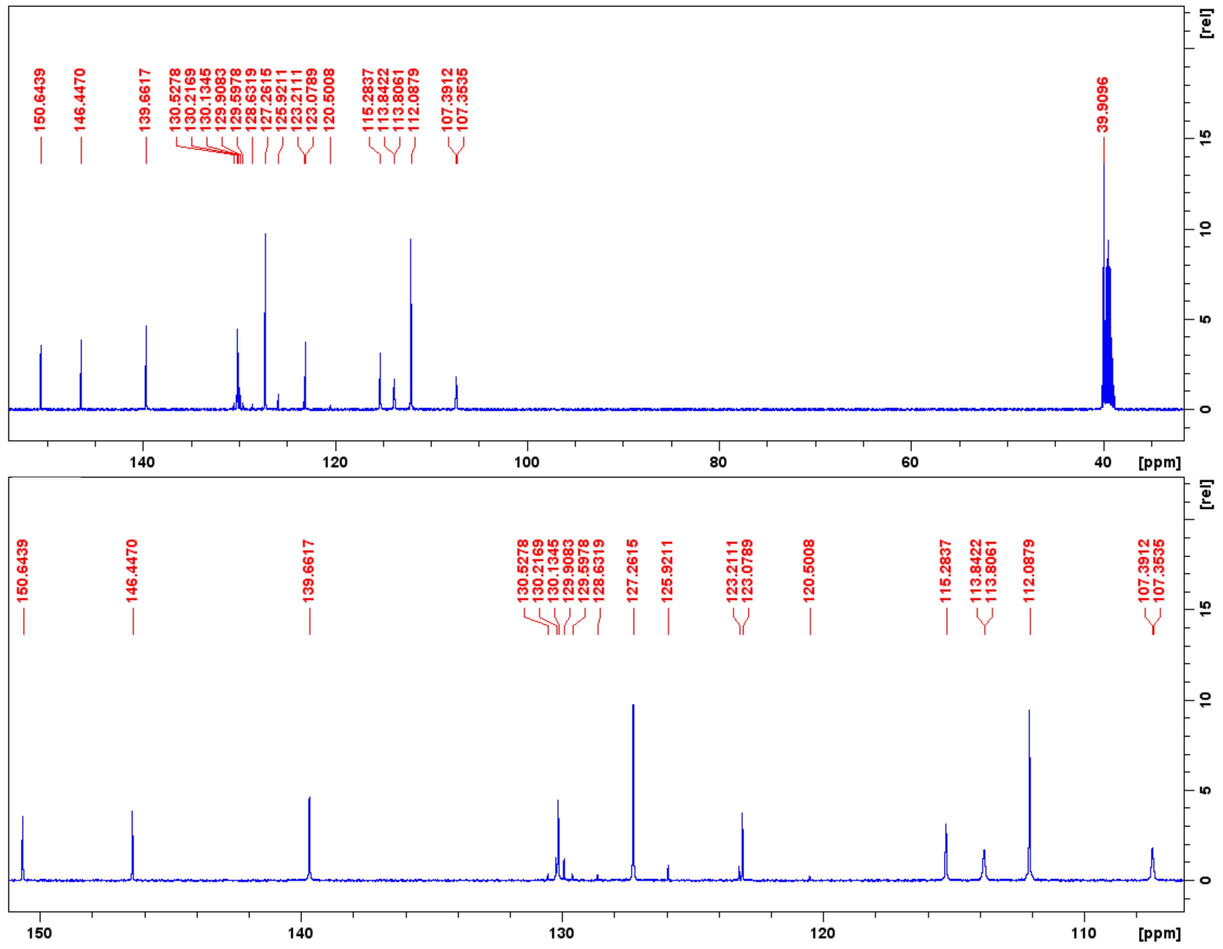

Figure S3 ^{13}C NMR of (*E*)-*N,N*-dimethyl-4-((2-(3-(trifluoromethyl)phenyl)hydrazinylidene) methyl) aniline (**HY-12**).

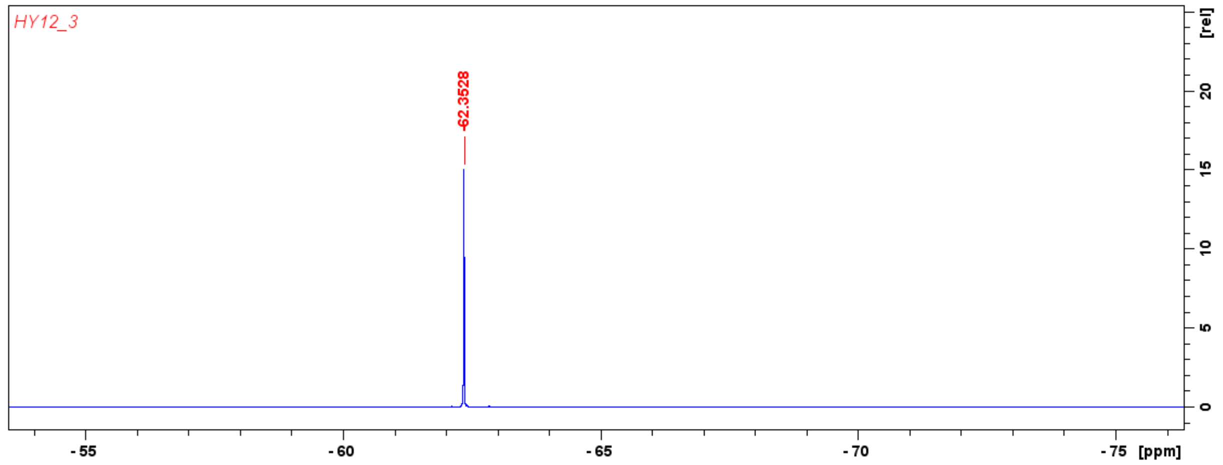

Figure S4 ^{19}F NMR of (*E*)-*N,N*-dimethyl-4-((2-(3-(trifluoromethyl)phenyl)-hydrazinylidene)-methyl) aniline (**HY-12**).

1.4. HPLC-MS traces of HY-12

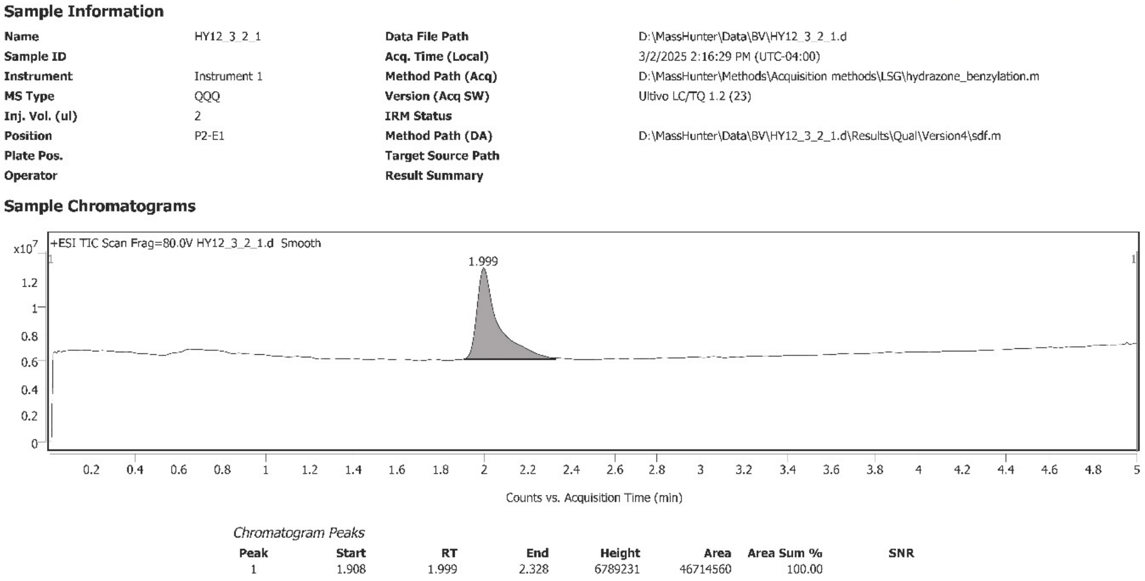

Figure S5 HPLC-MS trace of compound **HY-12**.

1.5 Human villous explant study

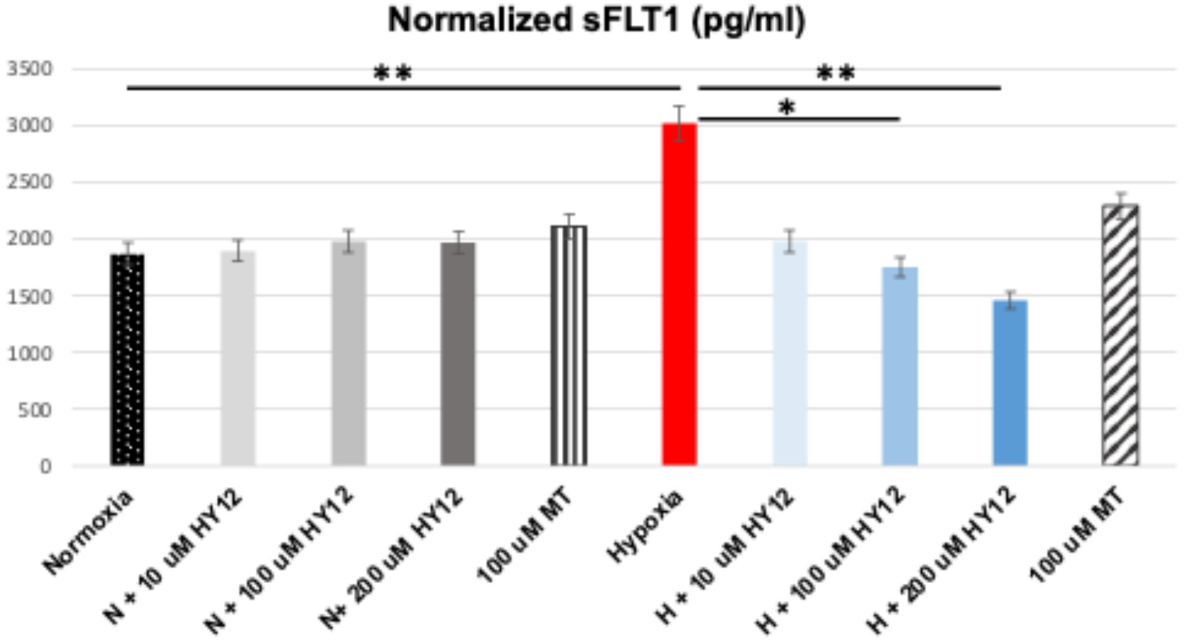

Figure S6 Compound HY-12 diminished sFLT-1 protein expression in in human villous explant culture during hypoxic exposure. Normalized sFLT1 data according to different

treatment groups (n=4 per group). Mann-Whitney-U-test. Median [IQR]. *: $p<0.05$. and **: $p<0.01$.

2. Biochemistry

2.1. Radical scavenging assays

HY-12 and the reference antioxidants (ascorbic acid and Trolox) were dissolved in dimethyl sulfoxide (DMSO) to a concentration of 100 mM and a serial dilution was then performed to obtain compound stock solution concentrations at 50 mM, 25 mM, 6.25 mM, 3.23 mM and 1.56 mM. The 2,2-diphenyl-1-picrylhydrazyl (DPPH) and 2,2'-azino-bis(3-ethylbenzo thiazoline-6-sulfonic acid (ABTS) assays were performed as described in our earlier article.[1]

2.1.1. DPPH radical scavenging assay

The DPPH assay was conducted as described earlier.[2] For the DPPH assay, the compound stock solutions were diluted in 37 °C 50% ethanol by a factor of 50. DPPH (Sigma-Aldrich, St. Louis, MO) was dissolved in 50 mL of 37 °C 50% ethanol to a concentration of 222 µM and stirred for 45 min in the dark to create the DPPH radical solution. On a clear flat bottom 96 well plate, 20 µL of each compound and 180 µL of DPPH were added in quadruplicate. Sets with 20 µL of 200 µM DMSO in 50% ethanol and 180 µL of 50% ethanol were used to determine the background absorbance. Sets with 20 µL of 200 µM DMSO in 50% ethanol and 180 µL of DPPH were used as the control group. The absorbance values were collected by a VersaMax UV-Vis plate reader that was set to 37 °C and 519 nm and processed with the SoftMax Pro 5 software (Molecular Devices). Readings were collected every 15 min for 60 min.

$$Percent\ Radical\ Scavenging = \frac{(Abs_c - Abs_t)}{Abs_c} \times 100 \qquad \text{(Equation S1)}$$

The 60 min values were standardized against the 60 min Trolox value to generate the Trolox equivalent values (Eq. 2).

$$Trolox\ Equivalence = \frac{Percent\ Radical\ Scavenging_{Sample}}{Percent\ Radical\ Scavenging_{Trolox}} \qquad \text{(Equation S2)}$$

2.1.2. ABTS radical scavenging assay

For the ABTS assay, 100 factor dilutions of the compound stock solutions were done in ethanol. The ABTS radical solution was prepared 16 to 24 h before the assay was started by dissolving ABTS (Tokyo Chemical Industry, Tokyo, Japan) and $K_2S_2O_8$ in 4 mL of deionized (DI) water to a concentration of 7 mM and 2.45 mM, respectively, and kept in the dark afterwards. The absorbance values were collected by a VersaMax UV-Vis plate reader that was set to 37 °C and 734 nm and processed with the SoftMax Pro 5 software (Molecular Devices). The ABTS radical solution was diluted with 37 °C 75 mM phosphate buffer with 75 mM NaCl at pH 7.4 until the absorbance was between 0.70 to 0.85 (the ratio was typically 5 µL of ABTS and 195 µL of phosphate buffer). On a clear flat bottom 96 well plate, 4 µL of each compound and 196 µL of ABTS were added in triplicate. Sets with 4 µL of 500 µM DMSO in ethanol and 196 µL of phosphate buffer determined background absorbance. Sets with 4 µL of 500 µM DMSO in ethanol and 196 µL of ABTS were used as the control group. Absorbance readings were taken at 0, 6, and 12 min.

The data from both assays were processed using following equation (Eq. S3), where Abs_c is the absorbance of the control and Abs_t is the absorbance of the test sample.

$$Percent\ Radical\ Scavenging = \frac{(Abs_c - Abs_t)}{Abs_c} \times 100 \qquad \text{(Equation S3)}$$

The 12 min value were standardized against the 12 min Trolox value to generate the final Trolox equivalent values (Eq.2).

3. Biological Evaluation

3.1. Cell culture studies: Human trophoblast HTR8/SVneo cell line was obtained from ATCC (American Type Culture Collection, Manassas, VA, USA). Cells were grown in RPMI supplemented with 5% fetal bovine serum and 1% penicillin-streptomycin in a humidified

incubator containing 5% CO_2 at 37 °C. Cells in the logarithmic growth phase were used in subsequent experimentation. Organofluorine antioxidant **HY-12** was dissolved in DMSO and stored at −20 °C until usage. The final concentration of DMSO in the medium was kept at less than 0.1% to not influence cellular health. Cells that received hydrogen peroxide received a dosage of 100 μM. Cells in the **HY-12** + H_2O_2-treated groups were pre-treated with 0.1-50 μM antioxidant for 30 minutes, respectively, and then they were treated with 100 μM H_2O_2 for 18 h in the media (containing hydrazones and hydrogen peroxide). At the end of the experiments, the culture supernatants were collected and stored at -20 °C until they were assayed.

3.2. Cell Viability Assay: A CCK-8 test (Cell Counting Kit-8, Dojindo Molecular Technologies, Inc. Shanghai, China), was used to measure cell viability following hydrogen peroxide and antioxidant treatments. HTR8/SVneo cells were plated in 48-well culture plates for the 24 hours prior to treatment. Following an 18-hour treatment with an antioxidant and H_2O_2, a total of 10 μL CCK-8 reagent was added in and incubated for 1 h in a 5% CO_2 incubator at 37 °C. After the incubation, optical density values were acquired at 450 nm using a microplate reader.

3.3. Cell biology measurements: HTR8/SVneo cells were seeded in 48-well plates (Nalgene Nunc International) and incubated at 37 °C in a 10% CO_2 humidified incubator overnight. Next day, cells received H_2O_2 treatment for 18 h, along with various concentrations of HY12 or reference antioxidant. After 18 h the cells were treated with MitoSOX™ Red (#M36008 Thermo Fisher Scientific) fluorogenic dye at 25 nM and 5 nM final concentrations, respectively, for 15 min. Cells underwent three PBS washes prior to dye visualization. Fluorescence of the various dyes were visualized and photographed using an inverted EVOS® FL Imaging System (Advanced Microscopy Group).

3.4. HIF1A measurement: Immunofluorescence was used to determine the nuclear translocation of HIF1A. HTR-8/SVneo cells were treated as mentioned previously.[3] Following treatment, the cells underwent three PBS washes followed by 4% paraformaldehyde fixation. Samples were then permeabilized with 0.5% Triton X-100, blocked with 1% BSA and then incubated with anti-HIF1A antibody (Alexa Fluor® 488 Anti-HIF1A antibody [EP1215Y] ABCAM#ab190197 1:200 dilution) overnight at 4 °C. DAPI was used to stain the nuclei. The coverslips were placed on glass

slides and samples were viewed under an inverted EVOS® FL Imaging System (Advanced Microscopy Group). Morphometric measurements of the trophoblasts were obtained through fluorescence microscopy images art 20x using MitoSOX™ Red and HIF1A and light microscopy images for trophoblasts COX EHC with an original magnification of 20x. Four images as replicates were used for analysis and measurements. Morphometric measurements were performed using ImageJ software version 1.47 (National Institute of Health [NIH], Bethesda, MD; http://imagej.nih.gov/ij). To determine staining intensity, the threshold was set to include the MitoSOX™ Red fluorescence product or 3,3'-diaminobenzidine (DAB) staining and the mean intensity (optical density; OD) of reaction product was calculated per image. The mean intensity was divided by cell area volume to calculate positivity per area.

3.5. Human Placental Villous Explant Cultures and Sample Collection: Placental samples were collected from patients with normotensive pregnancies and unlabored cesarian deliveries as described previously. The exclusion criteria for the current study of controls were maternal infection, diabetes mellitus, multiple gestations, kidney disease, and fetal anomalies. The Institutional Review Board at Beth Israel Deaconess Medical Center approved the collection and use of discarded human placentas.

For explant culture, individual clusters of villous trees were dissected under a stereomicroscope and cultured in 1 mL of DMEM/F12 media containing 10% fetal bovine serum (FBS) and 1% antibiotic-antimycotic (Gibco, Carlsbad, CA, USA). Fresh 0.3 M stock solutions of HY-12 were dissolved in DMSO and further diluted in DMEM/F12 media. The culture medium was supplemented with 10, 100, or 200 μM of HY-12 or Mito-TEMPO. MitoTEMPO stock solution was made at 10 mg/mL in sterile water and stored at −20 °C. The explants were maintained overnight at either 10% or 2% O2 with 5% CO2 at 37 °C.

3.6. sFLT-1 ELISA: Soluble FLT-1 (sFLT1) in culture medium was measured by ELISA using the human VEGF receptor 1 (VEGFR1) Quantikine kit (R&D Systems, Minneapolis, MN) following manufacturer's instructions and previously described.[3]

3.7. COX In situ enzyme chemistry: COX enzyme chemistry on HTR8/SVneo cells were performed as we published previously [1]. Representative digital images of cell preparations

(Thermo Scientific™: Nunc™ Lab-Tek™ II Chamber Slide™ System) were obtained. Four images were acquired and quantified per sample as replicates. Morphometric measurements were performed as described previously in HIF1A measurements.

3.8. Statistical analysis: GraphPad Prism 9.5 statistical software (San Diego, CA, USA) was used. After checking the normality tests (Kolmogorov-Smirnov, Saphiro-Wilk, D'Agostino & Pearson and Anderson-Darling tests), in case of normal distribution, parametric unpaired T-test or analysis of variance (ANOVA) with Tukey's post hoc test was used. Non-parametric Mann-Whitney-U test or Kruskal-Wallis test with Dunn's post hoc test was used when distribution was non-normal. Data are presented either in Mean ± Standard error of Mean (SEM) or in Median [Interquartile range/IQR]. Statistical significance was accepted when p-value was less than 0.05 ($p<0.05$).

4. References

1 W. Horton, S. Peerannawar, B. Török, M. Török, Theoretical and experimental analysis of the antioxidant features of substituted phenol and aniline model compounds. *Struct. Chem.* **2019**, *30*, 23–35.

2 S. Peerannawar, W. Horton, A. Kokel, F. Török, M. Török, B. Török, Theoretical and experimental analysis of the antioxidant features of diarylhydrazones. *Struct. Chem.* **2017**, *28*, 391-402.

3 D. Pintye *et al.*, Nitroxide-HMP-Protects Human Trophoblast HTR-8/SVneo Cells from H. *Antioxidants*, **2023**, *12*, 1578.
